# Using miRNA-Analyzer for the Analysis of miRNA Data

**DOI:** 10.3390/microarrays5040029

**Published:** 2016-12-15

**Authors:** Pietro Hiram Guzzi, Giuseppe Tradigo, Pierangelo Veltri

**Affiliations:** 1 Department of Surgical and Medical Sciences, University of Catanzaro, 88100 Catanzaro, Italy; 2 Dipartimento Informatica Modellistica ed Elettronica (DIMES), University of Calabria, 87036 Rende, Italy; gtradigo@dimes.unical.it (G.T.); veltri@unicz.it (P.V.)

**Keywords:** miRNA, data analysis, microarray

## Abstract

MicroRNAs (miRNAs) are small biological molecules that play an important role during the mechanisms of protein formation. Recent findings have demonstrated that they act as both positive and negative regulators of protein formation. Thus, the investigation of miRNAs, i.e., the determination of their level of expression, has developed a huge interest in the scientific community. One of the leading technologies for extracting miRNA data from biological samples is the miRNA Affymetrix platform. It provides the quantification of the level of expression of the miRNA in a sample, thus enabling the accumulation of data and allowing the determination of relationships among miRNA, genes, and diseases. Unfortunately, there is a lack of a comprehensive platform able to provide all the functions needed for the extraction of information from miRNA data. We here present miRNA-Analyzer, a complete software tool providing primary functionalities for miRNA data analysis. The current version of miRNA-Analyzer wraps the Affymetrix QCTool for the preprocessing of binary data files, and then provides feature selection (the filtering by species and by the associated *p*-value of preprocessed files). Finally, preprocessed and filtered data are analyzed by the Multiple Experiment Viewer (T-MEV) and Short Time Series Expression Miner (STEM) tools, which are also wrapped into miRNA-Analyzer, thus providing a unique environment for miRNA data analysis. The tool offers a plug-in interface so it is easily extensible by adding other algorithms as plug-ins. Users may download the tool freely for academic use at https://sites.google.com/site/mirnaanalyserproject/d.

## 1. Introduction

The understanding of function performed by genes requires the use of technologies to describe the gene’s behavior at a molecular level in a systematic way. Currently, among the existing technologies for the investigation of genes, microarrays are a well-established technology. The use of microarrays in research covers a broad range of disciplines. Starting from initial applications in genomics and molecular biology, more recently they found a large field of molecular medicine and clinical applications [1,2].

In particular, recent findings have attracted researchers on investigations of small molecules that play key roles on the complex mechanism of protein formation such as microRNAs. MicroRNA (miRNA) refers to a set of small RNA molecules composed of 21–23 nucleotides that do not encode any protein but participate as regulators in protein formation. Recent studies demonstrated that miRNA plays an essential role in carcinogenesis because the loss of their activity may affect the progression of the cell cycle, causing the development of tumor invasion and migration [3,4,5].

miRNAs are biomarkers for many therapies, and they are a molecular target therapy of many diseases. Therefore, the interest in studying miRNA behavior in clinical applications is increasing [6].

Thus, there is a need for the introduction of technologies to enable the study of miRNA expression in biological samples. Affymetrix provides one of the leading technologies for investigating such molecules, allowing the investigation of 15,644 different miRNAs of 131 known organisms [7].

A general-purpose workflow of miRNA data analysis comprises the following steps which may be composed of different methods depending on the microarray technology and the goals of the study:

- Loading of raw data from the use-specific libraries;
- Denoising data through preprocessing algorithms;
- Data-mining to extract knowledge;
- Interpretation of data by using ontologies.

In particular, raw data generated from Affymetrix platforms are initially preprocessed through the application of algorithms of summarization and normalization. The former class of algorithms tries to resume the raw value of miRNA expression by integrating different probesets corresponding to the same gene in a redundant fashion. The latter class of algorithms aims to mitigate the impact of noise by correcting the variation of miRNA expression values. Affymetrix provides a free tool, the QCTool, offering some algorithms for binary data management, summarization, and normalization (see Affymetrix documentation for further details www.affymetrix.com). Finally, probesets are filtered considering quality control criteria, e.g., the *p*-value associated with the reliability of the measurement that is provided by Affymetrix. Furthermore, the annotation process associates each miRNA to a set of functional information, e.g., as biological processes that are related to miRNA, and a set of pointers to available public databases [8,9].

Finally, statistical and data-mining analysis aims to infer biologically relevant knowledge by extracting the set of miRNAs that exhibit a consistent pattern of behaviors. Two examples of tools that provide such a function are the TMeV tool [10] and the STEM-miner tool [11].

Thus, researchers have to face different data formats, with data movement making the overall process time-consuming and error-prone. Thus, there is a need for the introduction of a comprehensive platform able to provide all the functions required for knowledge extraction from miRNA data.

Consequently, we designed miRNA-Analyzer, an extensible tool that provides the main functionalities for miRNA data analysis following the same principles underlying the development of the µ-CS [12,13] and DMET-Analyzer [14] tools. It can import raw Affymetrix data to provide some functions for data preprocessing, filtering and analysis. miRNA-Analyzer is also easily extensible through the addition of new plug-ins providing supplementary functionalities. The current version of miRNA-Analyzer wraps the Affymetrix Quality Control Tool (QC-Tool) for the preprocessing of binary data files; an ad hoc implemented filter for filtering by species and by associated *p*-values of preprocessed files; and the TmeV and STEM miner tools for the data analysis.

## 2. Materials and Methods

**Figure 1:**
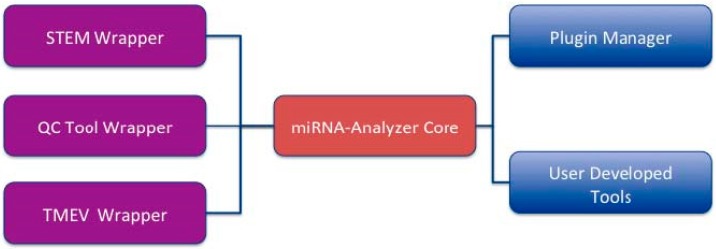
Architecture of miRNA-Analyzer.

**Figure 2:**
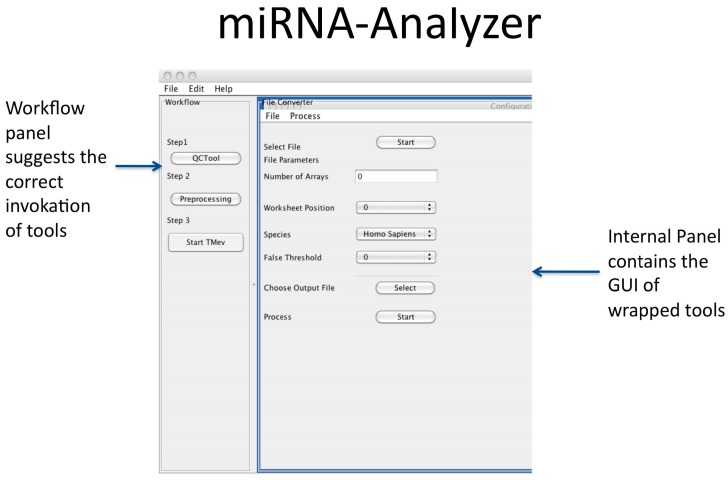
Graphical User Interface (GUI) of miRNA-Analyzer.

miRNA-Analyzer is based on Java Technology (www.java.com) and comprises following software modules (Figure 1 depicts the main software modules of miRNA Analyzer):

miRNA Analyzer Core: It represents the core of the application; it is responsible for the coordination of all the remaining modules by correctly invoking external modules and providing functionalities for data and files management. It is also responsible for interaction with users by offering a Graphical User Interface based on Java Swing as depicted in Figure 2.

Plugin Manager: Following a recent trend in bioinformatics, we decided to offer the possibility to the user to extend the application by designing and implementing novel plug-ins. For these reasons, we provide a plug-in management system. Such module is responsible for the management of plug-ins and defines application program interfaces (APIs) and interface for the development. Currently, the user may copy other stand-alone software modules written in Java in a specified folder of miRNA analyzer. Then the plug-in manager recognizes the modules, registers them in a registry and offers the possibility to invoke such modules to the users. Finally, the current release contains three main plug-ins that cover initial analysis functionalities.

STEM Wrapper: The STEM Miner tool is a well-known tool used for the analysis of short time series data. Since many published papers discuss the analysis of such kind of data, we decided to insert this tool into the core of our software by natively wrapping it.

T-MEV Wrapper: The T-MEV software platform provides a broad set of algorithms for data analysis (e.g., algorithms for clustering and classification) tailored for microarray data. Thus we decided to insert this tool into the core of our software by natively wrapping it.

QC-Tool Wrapper: The QC-Tool, as introduced before, is the Affymetrix provided software for initial data loading and pre-processing.

User Developed Tools: Users can develop another wrapper by following provided guidelines.

## 3. Results

We designed and implemented miRNA-Analyzer, a software tool allowing the semi-automatic summarization, annotation and analysis (classification, clustering, and time-series analysis) of Affymetrix binary data. It is based on a plugin-based architecture that allows increasing the functionalities by inserting novel plug-ins.

In particular, the current release wraps off-the-shelf preprocessing tools provided by Affymetrix, namely Affymetrix QC-Tools. Moreover, it implements an ad hoc designed feature selection module that enables the selection of a subset of probesets based on quality data calculated on raw images (such as the detection of *p*-values).

Finally, it comprises a plugin management system that enables the insertion of analysis functionalities provided by another tool. Currently, software tools based on Java (www.java.com) technology may be directly integrated into miRNA by drag-and-drop, while for other instruments the user has to write an ad hoc Java class following specified recommendations.

## 4. Conclusions

We presented miRNA-Analyzer, a software tool that provides the main functionalities for miRNA data analysis. It is based on an extensible software architecture based on the wrapping of some existing modules such as the Affymetrix QC-Tool for the preprocessing of binary data files, the TmeV and STEM miner tools for analysis, and some ad hoc developed modules for data filtering. The tool offers a plug-in interface so it is easily extensible by adding other algorithms as plug-ins.
